# COVID-19 and Mental Well-Being: Guidance on the Application of Behavioral and Positive Well-Being Strategies

**DOI:** 10.3390/healthcare8030336

**Published:** 2020-09-12

**Authors:** Amar Kanekar, Manoj Sharma

**Affiliations:** 1 School of Counseling, Human Performance & Rehabilitation, Health Education/Health Promotion, University of Arkansas at Little Rock, Little Rock, AR 72204, USA; 2 Environmental & Occupational Health, School of Public Health, University of Nevada, Las Vegas, NV 89119, USA; manoj.sharma@unlv.edu

**Keywords:** COVID-19, mental well-being, coping, stress reduction

## Abstract

The raging COVID-19 pandemic has been a great source of anxiety, distress, and stress among the population. Along with mandates for social distancing and infection control measures, the growing importance of managing and cultivating good mental well-being practices cannot be disregarded. The purpose of this commentary is to outline and discuss some research-proven positive well-being and stress reduction strategies to instill healthy coping mechanisms among individuals and community members. The authors anticipate that usage of these strategies at the individual and the community level should greatly benefit the mental well-being not only in the current COVID-19 pandemic but also in any future epidemics at the national level.

## 1. Introduction

COVID-19 is an unprecedented pandemic affecting people all over the world. As of 29 August 2020, COVID-19 (caused by the novel coronavirus) has caused 5,845,876 cases and 180,165 total deaths in the United States [1] and 24,257,989 cases and 827,246 deaths worldwide [2]. The pandemic is still raging havoc at the time of writing this article. Pandemic by definition means when a disease or a condition spreads across countries and continents [3].

The COVID-19 pandemic with its uncertainty has imposed great mental distress on the general public, its patients, and healthcare providers [4]. The pandemic and its constant reporting in the media have increased distress-related psychological problems such as anxiety, depression, and insomnia [5,6]. At present, there is no established treatment for COVID-19 or any vaccine for specific protection against it. The testing for COVID-19 is not widely available and lacks desirable sensitivity and specificity [7,8]. The testing of its antibodies is also not quite accurate or readily available. Hence, the current public health measures include preventing person-to-person transmission of the disease by separating people. Among the approaches that are being used are (1) isolation in which infected persons are separated from non-infected individuals; (2) quarantine and fever surveillance of contacts who have been exposed but are not yet symptomatic; (3) community containment in which social distancing and movement of the general public is restricted by efforts such as “stay at home orders” (community-wide quarantine) [9]. Such measures further compound the emotional distress being experienced by individuals. The pandemic also has an important economic aspect to it with millions of people losing their employment, which is a great source of emotional distress [10,11].

The fear associated with this pandemic is responsible for the activation of the hypothalamus-pituitary-adrenal (HPA) axis [12]. The hypothalamus liberates the corticotrophin-releasing hormone (CRH) in response to emotional distress, which in turn, activates the pituitary gland to liberate the adrenocorticotropic hormone causing the liberation of cortisol from the adrenal cortex. Cortisol, a glucocorticoid hormone, affects the body in several ways. For example, it affects the sleep/wake cycle, it affects the glucose metabolism, it regulates the blood pressure, and it boosts energy so one can handle stress [13]. All these effects eventually drain the body’s energy resources in the long run and also compromise immunity and mental resilience [14].

Although the Centers for Disease Control and Prevention (CDC) have provided some guidelines to reduce stress and initiate coping [9], the need of the hour seems to be planning and having resources and techniques for long-term mental health flourishing and better emotional health management. Recent reports from the World Health Organization calls for global action to invest in and strengthen mental health services to avert an impending mental health crisis [15].

Mental health denotes emotional, psychological, and social well-being [16]. Positive mental health and positive psychology have an imminent role to play during this unprecedented public health crisis. Although there is enough evidence-based literature on the application of positive mental health techniques at individual level for stress reduction or life fulfillment, its application in a pandemic scenario is minimally explored [17,18].

The purpose of this commentary is to address the unexpected and uncertain situation experienced due to this pandemic (which is to cause anxiety, alarm, panic) and a deep sense of ongoing fear by providing readers with research-proven techniques and strategies for generating and maintaining momentary and lifelong happiness, fulfillment, and entitlement to positive being and positive living. Some of the strategies such as nurturing and maintaining social connections (while maintaining physical distancing), mindfulness and momentary living, goal commitment, and resilience [19] will be explored, particularly from its applicability to the current COVID-19 pandemic. The authors will additionally explore the science of gratitude development and maintenance as a strong strategy in this pathway.

## 2. Happiness and Mental-Well-Being Strategies

Happiness strategies classically outlined in Lyubomirsky’s book “The How of Happiness” revolve around (a) living in the present, (b) managing stress (which is outlined later in this article), and (c) investing in social connections [19]. Similarly outline strategies of broadening your thinking, raising your positivity-ratio, and disputing negative thinking and fear (which is obvious during pandemics) greatly assist in maintaining well-being at its highest levels [20].

Mindfulness meditation practice daily helps in quieting one’s mind and prevents the constant internal mental chatter. This is additionally proven to focus your attention on the present moment and a lot of existing research has proven the efforts of its practice in maintaining and nurturing improved mental health. [21,22]. For example, the student population has greatly benefitted from a mindfulness course in terms of improved well-being, decreased stress, and increased resilience [23]. Similar benefits were noted in diverse populations such as older adults [24], adolescents [25], and educators [26]. A systematic review [27] and another meta-analysis [28] found that mindfulness-based stress reduction (MBSR) was effective in reducing stress, depression, anxiety, and distress and in improving the quality of life of healthy individuals.

The role of religion and prayer in reducing stress cannot be overemphasized such that studies have proven that prayer plays a significant role which is no less than meditation and other mind–body techniques in reducing stress [29]. Social connections (some of which are explained later) have shown proven associations between long-term well-being [30,31,32], and this could be practiced in a ‘lockdown’ environment by way of telephonic, message, and video-contact with family, friends, and colleagues.

## 3. Gratitude Science and Building Gratitude for Positive Mental Health

The role of dispositional and/or trait gratitude in mental well-being is a comparatively recent development in positive psychology [33,34]. There are some possible mechanisms of applying gratitude for generating positive mental well-being leading to prolonged life satisfaction and flourishing in life such as: (a) savoring positive life experiences, particularly in eras of pandemics such as spending time with your kids, having healthy meals or pursuing hobbies while being indoors could be joyful; (b) building positive emotions, which help in creative activities such as writing, playing an instrument, painting, and singing, and finally (c) engaging in social connections via electronic means and video-chats with family and friends, which assist in generating social bonds leading to improved relationships, elevated self-esteem, and overall psychological well-being [35]

Living indoors and not going outdoors due to ‘lockdowns’ in a pandemic provides an individual level opportunity for self-introspection and assessing and reframing current and planned events through a positive lens and engaging in active problem solving [34]. Building and maintaining gratitude through actions of kindness, being thankful that one is living, and enjoying all the benefits that life offers also helps in coping with stressful situations by building lifelong resilience [35].

## 4. Application of Behavioral Theories in Promotion of Mental Well-Being

There are several determinants of positive mental health such as hardiness, sense of coherence, social support, optimism, and self-esteem [36] that are important in the context of COVID-19. According to the hardiness theory [37], three attributes can enhance our coping. The first one is “control” that pertains to one’s belief that one can influence the environment. In the case of COVID-19, the control can come from taking all the precautionary measures that are under one’s control. If one has lost his or her job, one needs to still maintain a sense of control and continue trying for alternatives. Adhering to such measures will help one endure the adverse effects of distress and have better mental health. The second attribute in hardiness is that of “commitment”, which pertains to one’s deep involvement in whatever one does. With COVID-19, if one is confined to the home one can get involved in creative activities such as writing, cooking, drawing, and other activities that keep one busy. Searching for a job if one has lost one’s job with commitment will also lower distress. Such commitment to everyday activities will help cope with stress and achieve better mental health. The third and final attribute of hardiness is that of “challenge”, which pertains to one’s ability to undertake change, confront new activities, and seek avenues for growth. The COVID-19 pandemic provides ample opportunity for the challenge, which if harnessed appropriately, can foster positive mental health.

The second theory that is of relevance to COVID-19 is the sense of coherence theory [38,39]. The three components of the sense of coherence are comprehensibility, manageability, and meaningfulness. Comprehensibility pertains to the ability to see the stressor that one faces as making some sense in the context of its structure, consistency, order, clarity, and predictability. With COVID-19, the comprehensibility of the stressor is lost and replaced with uncertainty, which results in distress. The second component of manageability pertains to the ability to believe that the resources under one’s control are sufficient to meet the demands posed by the stressors. With COVID-19, one may at times feel that one is overwhelmed, but once again reminding oneself that the problem is temporary and the solution is inbuilt in the problem will go a long way in lowering distress and fostering positive mental health. The final aspect of the sense of coherence is that of meaningfulness, which pertains to the belief that life makes sense, and that the stressors in life are worthy of putting efforts into dealing with. It requires accepting stressors in life as challenges instead of feeling that they are burdensome. This type of attitude in dealing with COVID-19-related crises of any kind is vital not only in dealing with emotional distress but also in succeeding in life.

The third theory that is of relevance in the COVID-19 pandemic is that of social support, which is the help obtained through social relationships [40,41]. Social support was classified into four kinds: (1) emotional support that requires the provision of understanding, caring, love, and fosters reliance; (2) informational support that requires the provision of information, counsel, and guidance; (3) instrumental support that requires the provision of tangible help; (4) appraisal support that provides evaluative help. During these times of COVID-19 pandemic, all these types of social support are very much needed. One needs emotional support to buffer emotional distress; one needs informational support to keep abreast with latest developments on the disease, resources, and opportunities; one needs instrumental support in the form of tangible resources, and one needs appraisal support on various facets of dealing with the pandemic and its influence on one’s life.

Another theory linked to good mental health is that of optimism [42], which requires one to expect the best possible outcome in any situation and is a learned behavior [43]. Optimism, in the COVID-19 context, will operate through enhancing one’s efforts to avoid the disease by increasing one’s attention to information regarding its threat, directly improving coping, and building a positive mood.

A final theory that is popular in the mental health field and common parlance is that of self-esteem [44,45]. A favorable attitude of oneself or confidence in one’s self-worth is very important for mental health and must be maintained during the COVID-19 pandemic no matter what the circumstances are.

## 5. ‘Death Anxiety’ and the COVID-19 Pandemic

The concept of ‘death anxiety’—the anxiety and psychological distress among human beings due to thoughts related to fear of death in the current COVID-19 pandemic—has been growing recently such that ‘coronaphobia’ has been quite evident as a construct predicting generalized anxiety along with death anxiety across the population [46]. Fortunately, this anxiety can be measured [47] and techniques used to manage it. Although some of the strategies as suggested by the World Health Organization such as minimizing news feeds and promoting social media usage could be beneficial [48], emerging research suggests the role of positive self-talks and cognitive behavior therapy as effective modalities to modify or attenuate the ‘death anxiety’ [49,50].

Thought interference, particularly annoying thoughts related to fear of death due to COVID-19 can be very disturbing for individuals, and these strategies promote the ‘problem-centered’ coping style [51] for stress reduction and, along with the behavioral strategies mentioned earlier, could be highly effective.

## 6. Conclusions

The COVID-19 pandemic continues to dominate the public health field. The authors believe that although the initial panic caused by the pandemic has mitigated to some extent its effects (such as anxiety, stress, fear, and uncertainty) will continue to linger for months ahead.

There were a number of theories discussed in this commentary such as the hardiness, sense of coherence, and the social support theory. These theories when applied to a pandemic scenario, such as the current COVID-19 scenarios, greatly helps us shape our understanding of the impact that this pandemic is having on anxiety, fear, and stress. Social support theory guides us in managing and coping with these mental health conditions. Future research should be aimed at the application of these theories in improved understanding of the role they play specifically in the COVID-19 pandemic, and how the constructs of these theories could be modified to enhance mental health and well-being among COVID-19 affected individuals. Social support theory-based constructs could be utilized in developing and implementing interventions in preventing and promoting mental health in COVID-19 affected individuals.

Additionally, the behavioral and positive well-being strategies outlined and discussed in this commentary provide guidance not only to individuals and community members at the frontline of this pandemic but also to people staying at home due to ‘stay at home’ orders. It behooves us to make use of as many behavioral strategies in our repertoire in these unprecedented and precarious times.
